# An open-label study to evaluate the long-term safety and efficacy of lanadelumab for prevention of attacks in hereditary angioedema: design of the HELP study extension

**DOI:** 10.1186/s13601-017-0172-9

**Published:** 2017-10-06

**Authors:** Marc A. Riedl, Jonathan A. Bernstein, Timothy Craig, Aleena Banerji, Markus Magerl, Marco Cicardi, Hilary J. Longhurst, Mustafa M. Shennak, William H. Yang, Jennifer Schranz, Jovanna Baptista, Paula J. Busse

**Affiliations:** 1 University of California – San Diego School of Medicine, 8899 University Center Lane, Suite 230, San Diego, CA 92122 USA; 2 Department of Internal Medicine/Allergy Section Cincinnati, University of Cincinnati College of Medicine, 231 Albert Sabin Way, ML#563, Cincinnati, OH 45267 USA; 3 Department of Medicine and Pediatrics, Penn State University, Allergy, Asthma and Immunology, 500 University Drive, Hershey, PA 17033 USA; 4 Division of Rheumatology, Allergy and Immunology, Department of Medicine, Massachusetts General Hospital, Harvard Medical School, 55 Fruit Street, Cox 201, Boston, MA 02114 USA; 5 Department of Dermatology and Allergy, Charité–Universitätsmedizin Berlin, Charitéplatz 1, 10117 Berlin, Germany; 6 Department of Biomedical and Clinical Sciences, Luigi Sacco, University of Milan, ASST Fatebenefratelli-Sacco Milan, Via G.B. Grassi 74, 20157 Milan, Italy; 7 Department of Immunology, Barts Health NHS Trust, 80 Newark Street, London, E1 2ES UK; 8 Triumpharma Inc., 07 Building, Al Yarooty Street, PO Box 2233, Amman, 11941 Jordan; 9 Ottawa Allergy Research Corporation, University of Ottawa Medical School, 110-2935 Conroy Road, Ottawa, ON K1G 6C6 Canada; 10 Shire, 300 Shire Way, Lexington, MA 02421 USA; 11 Division of Clinical Immunology and Allergy, Department of Medicine, Icahn School of Medicine at Mount Sinai, 5 East 98th Street 11th Floor, New York, NY 10029 USA

**Keywords:** Hereditary angioedema, Lanadelumab, Monoclonal antibody, Bradykinin, Plasma kallikrein, Prophylaxis, C1-inhibitor, Rare disease, Orphan disease

## Abstract

**Background:**

Hereditary angioedema (HAE) is characterized by recurrent attacks of subcutaneous or submucosal edema. Attacks are unpredictable, debilitating, and have a significant impact on quality of life. Patients may be prescribed prophylactic therapy to prevent angioedema attacks. Current prophylactic treatments may be difficult to administer (i.e., intravenously), require frequent administrations or are not well tolerated, and breakthrough attacks may still occur frequently. Lanadelumab is a subcutaneously-administered monoclonal antibody inhibitor of plasma kallikrein in clinical development for prophylaxis of hereditary angioedema attacks. A Phase 1b study supported its efficacy in preventing attacks. A Phase 3, randomized, double-blind, placebo-controlled, parallel-arm study has been completed and an open-label extension is currently ongoing.

**Methods/design:**

The primary objective of the open-label extension is to evaluate the long-term safety of repeated subcutaneous administrations of lanadelumab in patients with type I/II HAE. Secondary objectives include evaluation of efficacy and time to first angioedema attack to determine outer bounds of the dosing interval. The study will also evaluate immunogenicity, pharmacokinetics/pharmacodynamics, quality of life, characteristics of breakthrough attacks, ease of self-administration, and safety/efficacy in patients who switch to lanadelumab from another prophylactic therapy. The open-label extension will enroll patients who completed the double-blind study (“rollover patients”) and those who did not participate in the double-blind study (“non-rollover patients”), which includes patients who may or may not be currently using another prophylactic therapy. Rollover patients will receive a single 300 mg dose of lanadelumab on Day 0 and the second dose after the patient’s first confirmed angioedema attack. Thereafter, lanadelumab will be administered every 2 weeks. Non-rollover patients will receive 300 mg lanadelumab every 2 weeks regardless of the first attack. All patients will receive their last dose on Day 350 (maximum of 26 doses), and will then undergo a 4-week follow-up.

**Discussion:**

Prevention of attacks can reduce the burden of illness associated with HAE. Prophylactic therapy requires extended, repeated dosing and the results of this study will provide important data on the long-term safety and efficacy of lanadelumab, a monoclonal antibody inhibitor of plasma kallikrein for subcutaneous administration for the treatment of HAE.

*Trial registration* NCT02741596

**Electronic supplementary material:**

The online version of this article (doi:10.1186/s13601-017-0172-9) contains supplementary material, which is available to authorized users.

## Background

Hereditary angioedema (HAE) is a rare genetic disorder that affects an estimated 1 in 50,000 individuals [1]. It is characterized by recurrent debilitating attacks of subcutaneous or submucosal edema, most often affecting the face, gastrointestinal tract, extremities, genitalia, and larynx [2]. Patients experience symptoms of localized swelling and pain. HAE type I and type II are caused by a deficiency in the quantity or functional activity of C1 inhibitor (C1-INH), which results in dysregulation of plasma kallikrein activity and excessive release of the vasodilator bradykinin from high molecular weight kininogen (HMWK) [1, 3].

As angioedema attacks are unpredictable, and those affecting the larynx can be life-threatening, one treatment strategy for HAE involves the use of prophylactic agents to prevent angioedema attacks. Current long-term prophylactic therapies include the replacement of C1-INH with plasma-derived C1-INH or treatment with attenuated androgens (Table 1). However, C1-INH replacement requires frequent intravenous administration, which may be inconvenient and difficult for some patients, and androgens are limited by a poor safety profile, especially for female and pediatric patients [1, 4].

**Table 1:** Current therapeutics for long-term prophylaxis in HAE

Prophylactic treatment	Route of administration	Dosing frequency	Efficacy	Safety
Plasma-derived C1-INH	Intravenous	Every 3–4 days	Effective in reducing attack rate [17, 18] Breakthrough attacks still reported [17, 18]	Risk of thromboembolic events [19] No safety concerns in patients treated with up to 2500 U [20]
Androgens	Oral	Daily	Treatment associated with significantly fewer and less severe attacks [4] Breakthrough attacks still reported [21]	Most common side effects: weight gain, virilization, headaches, myalgia, mood changes, menstrual disorders, liver dysfunction, increased serum lipids [4] Contraindicated in pediatrics and during pregnancy [1]
Tranexamic acid	Oral	Daily	Not licensed for long-term prophylaxis, although often used for this indication [1] Efficacy unconfirmed. In 12 patients with HAE, 50% had some improvement in HAE attack rate [22]	Gastrointestinal upset, myalgia, theoretical risk of thrombosis [1]

*C1*-*INH* C1 inhibitor, *HAE* hereditary angioedema

Lanadelumab (DX-2930) is a fully human monoclonal antibody inhibitor of plasma kallikrein. Inhibition of plasma kallikrein is an attractive and rational therapeutic strategy for HAE as it prevents the production of bradykinin. Lanadelumab is a highly potent (K_i_ = 125 pM), specific, and rapid inhibitor of plasma kallikrein that was discovered by phage display technology and is produced in Chinese Hamster Ovary cells using standard methods [5]. It received fast-track and breakthrough therapy designations from the US Food and Drug Administration and is currently in Phase 3 clinical development for prophylaxis against HAE attacks. Data from two Phase 1 studies did not identify any safety concerns (Table 2) [6, 7]. A Phase 1b study in patients with HAE treated with two subcutaneous doses of 300 and 400 mg lanadelumab suggested efficacy in preventing angioedema attacks [7].

**Table 2:** Safety and efficacy results from Phase 1a and 1b studies with lanadelumab

Study	Population	N	Treatments	TEAEs	Anti-drug antibodies	Efficacy
Phase 1a^a^	Healthy subjects	32	Single dose: Placebo 0.1, 0.3, 1.0, or 3.0 mg/kg lanadelumab	Most common TEAE was headache (25% of both placebo- and lanadelumab-treated patients)	None	NA
Phase 1b^b^	Patients with HAE	37	Two doses: Placebo 30, 100, 300, or 400 mg lanadelumab	Most common treatment emergent adverse events for lanadelumab versus placebo were angioedema attacks (38 vs 69%), injection site pain (25 vs 23%), and headache (17 vs 23%) Local AEs in 25 vs 23.1% for lanadelumab and placebo, respectively 3 severe related treatment emergent adverse events: injection site pain (1 patient); headache, night sweats (1 patient)	Positive results in 3/92 (3.3%) post-dose samples from 2/23 patients (8.7%) None were neutralizing	Angioedema attack rate decreased by 100% in 300 mg group (P < 0.0001) and by 88% in 400 mg group (P = 0.005)

*AE* adverse event; *HAE* hereditary angioedema; *NA* not applicable; *TEAE* treatment-emergent adverse event

^a^DX-2930-01 (NCT01923207) [6]

^b^DX-2930-02 (NCT02093923) [7]

A pivotal, Phase 3, multicenter, randomized, double-blind, placebo-controlled study (HELP Study [DX-2930-03; NCT02586805]) enrolling up to 120 patients to further assess the safety and efficacy of lanadelumab has been completed [8]. Patients who completed the double-blind study were offered the option of continuing into the long-term open-label extension (HELP Study Extension [DX-2930-04; NCT02741596]) [9]. In addition, the open-label extension study will enroll 100 patients who did not participate in the double-blind study and who may or may not be currently receiving prophylactic treatment. The open-label extension study will evaluate the long-term safety of subcutaneously administered lanadelumab (over 12–18 months of exposure across both studies) and its long-term efficacy in preventing angioedema attacks.

## Methods/design

### Objectives

The primary objective of this study is to evaluate the long-term safety of repeated subcutaneous administrations of lanadelumab in patients with HAE.

Secondary objectives include evaluation of the long-term efficacy of lanadelumab in preventing HAE attacks and the outer bounds of dosing frequency (i.e., the duration of its clinical effect) by assessing the length of time between a rollover patient’s first open-label dose of lanadelumab and their first reported HAE attack.

Tertiary objectives include evaluation of immunogenicity, health-related quality of life, the pharmacokinetic/pharmacodynamic profile of lanadelumab, safety and efficacy associated with switching from another long-term prophylactic therapy to lanadelumab, characteristics of breakthrough attacks compared with historical baseline, and experience with self-administration of lanadelumab.

### Study setting

A total of 43 study sites are planned across North America, Europe, and the Middle East. Patients may, after receiving the first two doses of study drug at the site and after completing training, self-administer lanadelumab off site.

### Study design

The open-label extension study will consist of patients who complete the double-blind study (rollover patients) and additional patients who are not part of the double-blind study (non-rollover patients). All patients will receive open-label lanadelumab every 2 weeks over a 364-day treatment period, followed by a 4-week safety follow-up before discharge from the study.

### Study patients

**Fig. 1:**
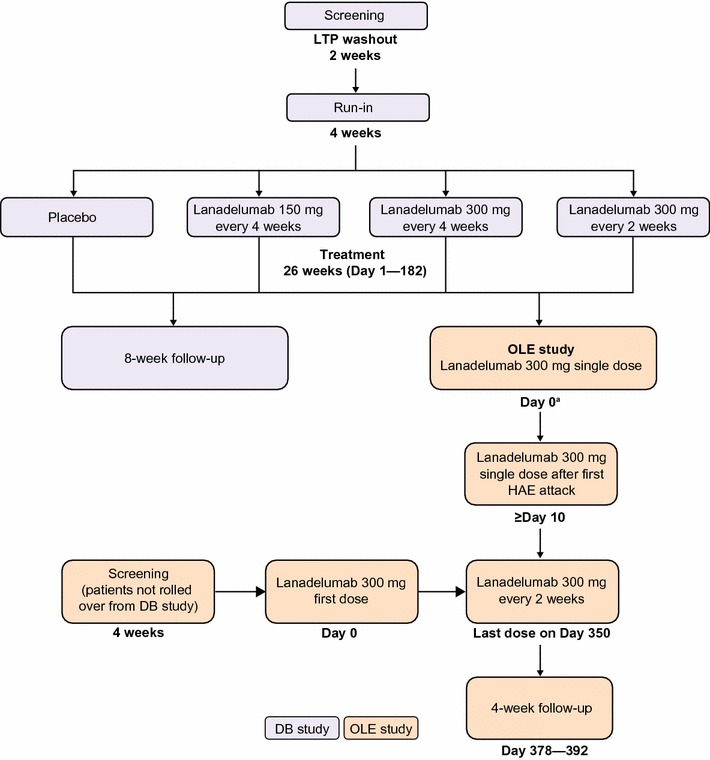
Overview of study design. *DB* double-blind; *HAE* hereditary angioedema, *LTP* long-term prophylaxis; *OLE* open-label extension. ^a^For rollover patients, Day 0 of the open-label extension study coincides with Day 182 of the double-blind study

All patients enrolled in the double-blind study will be eligible for rollover into the open-label extension study (Fig. 1). Patients who discontinue from the double-blind study after enrollment will not be eligible to enroll in the open-label extension study.

Up to 100 non-rollover patients will also be screened and enrolled in the open-label extension study. Among these will be patients who are using another long-term prophylactic agent in order to evaluate the efficacy and safety of switching from another prophylactic therapy to lanadelumab. Patients who did not qualify for the double-blind study may screen for eligibility in the open-label extension.

The study will aim to enroll at least 15 patients who are 12–17 years of age.

### Inclusion criteria

Patients must meet the following criteria to be enrolled in the study:

1. Male or female patients who are ≥ 12 years of age at the time of screening.
2. Patients must have documented confirmation of type I/II HAE. Confirmation will require (1) a clinical history consistent with HAE, (2) diagnostic testing results that confirm HAE (C1-INH functional level < 40% of normal. Patients with functional C1-INH at 40–50% of normal may be enrolled if they also have a C4 level below the normal range), and (3) either age of onset of first angioedema symptoms ≤ 30 years, a family history consistent with HAE, or C1q within normal range.
3. Patients must have a historical baseline attack rate of at least one attack per 12 weeks.
4. Patients and/or their caregivers (as appropriate) must be able to provide informed consent or assent (as appropriate).
5. Patients must adhere to contraception requirements for the duration of the study.

### Exclusion criteria

Patients who meet any of the following will be excluded from the study:

1. Discontinued from the double-blind study for any reason.
2. If rolling over from the double-blind study, the presence of any safety concerns that would preclude participation in the open-label extension study.
3. Concomitant diagnosis of another form of chronic recurrent angioedema such as acquired angioedema, HAE with normal C1-INH, idiopathic angioedema, or recurrent angioedema associated with urticaria.
4. Exposure to an investigational drug (excluding lanadelumab or other HAE therapies) or investigational device within 4 weeks prior to screening.
5. Exposure to angiotensin-converting enzyme inhibitors within 4 weeks prior to screening or exposure to any newly initiated or modified dose of systemic estrogen-containing medications within 3 months prior to screening.
6. Unwilling to discontinue use of long-term prophylaxis (C1-INH, androgens or anti-fibrinolytics) within 3 weeks after starting lanadelumab treatment.
7. Presence of liver function abnormalities.
8. Pregnant or breastfeeding.
9. Presence of any condition that, in the opinion of the investigator or sponsor, may compromise the patient’s safety or compliance, preclude successful conduct of the study or interfere with interpretation of the results.

### Interventions

Patients will receive open-label 300 mg (2 mL) subcutaneous injections of lanadelumab every 2 weeks, as this dose is anticipated to be effective and safe. If at any time an important dose-related safety signal is identified from either the double-blind or the open-label extension study, patients who have not completed the study and any subsequent patients may be switched to a different dose and/or dosing frequency. In addition, a different dose and/or frequency may be used based on the efficacy and safety results of the double-blind study.

For rollover patients, a single dose of lanadelumab will be administered on Day 0 of the open-label extension study (coinciding with Day 182 of the double-blind study). The second dose of lanadelumab will not be administered until after the first investigator-confirmed angioedema attack. The day of the second dose will vary depending on when the first attack occurs, but there must be a minimum of 10 days between the Day 0 dose and the second dose. Thus, patients who experience an attack within 10 days of the first dose will not receive the second dose until Day 10. Thereafter, 300 mg lanadelumab will be administered every 2 weeks, with the final study dose administered on Day 350, for a maximum of 26 doses. All patients, caregivers, investigators, and study site personnel will remain blinded to the double-blind treatment assignment until the conclusion of the open-label extension study.

Non-rollover patients will receive their first open-label 300 mg dose of lanadelumab on Day 0, with dosing for the study continuing every 2 weeks until Day 350, regardless of the first attack, for a total of 26 doses.

Patients who are considered suitable candidates may qualify to self-administer lanadelumab after the second dose. Patients must complete appropriate training for self-administration and demonstrate the comprehension and technique to self-administer.

### Concomitant therapies

Therapies for coexisting conditions, including the treatment of angioedema attacks and short-term prophylaxis, are permitted as described below.

Long-term prophylaxis will not be permitted once it has been discontinued, as described below. Androgens may not be used for HAE or for any medical condition. Use of angiotensin-converting enzyme inhibitors, estrogen-containing medications with systemic absorption, and any other investigational drug or device is not permitted, as described under *Exclusion criteria*.

### Current prophylactic therapies

In the absence of formal guidelines for withdrawal of long-term prophylaxis, our approach is based on clinical expert recommendations. Current long-term prophylactic therapies will be tapered off. Use of C1-INH may continue until Day 15. Androgens or anti-fibrinolytics may also be used up to Day 15 but, if necessary, may be extended until a maximum of 3 weeks after the first lanadelumab dose.

The use of C1-INH as a short-term (pre-procedure) prophylactic treatment for HAE will be permitted if medically indicated.

### Management of angioedema attacks

Angioedema attacks that occur during the study will be managed in accordance with the investigator’s usual standard of care, including use of acute therapies that the investigator deems to be medically appropriate. Use of C1-INH will be permitted for treatment for an angioedema attack. Administration of lanadelumab and study procedures will continue as planned in the protocol, even if the patient has symptoms of an angioedema attack the day of lanadelumab administration and/or receives treatment for an angioedema attack.

### Outcomes

All study activities are shown in Additional file 1: Table S1.

Safety assessments will include the following:

- Adverse events, including serious adverse events and adverse events of special interest, such as hypersensitivity reactions and events of disordered coagulation (bleeding, hypercoagulation). HAE attacks that occur during the study will be captured as adverse events
- Vital signs (blood pressure, heart rate, body temperature, respiratory rate)
- Clinical laboratory tests (hematology, serum chemistry, coagulation, urinalysis)
- Physical examination
- 12-lead electrocardiogram.

The following information will be collected for HAE attacks that occur during the study:

- Date and time the attack symptoms started
- Description of symptoms experienced, including location(s)
- Impact on activity (work, school, social interactions)
- Assistance or medical intervention required (hospitalizations, additional laboratory tests, emergency department visits)
- Any medications used to treat the attack (both prescription and over-the-counter)
- If the attack resolved, date and time of symptom resolution.

Patients or their caregivers must notify and report details to the study site within 72 h of the onset of an angioedema attack. If desired, a memory aid such as a diary may be used to assist in tracking HAE attacks. To be confirmed as an angioedema attack, the event must have symptoms or signs consistent with an angioedema attack. The investigator or physician designee will clinically determine whether the event did or did not represent an angioedema attack. To be counted as a unique angioedema attack distinct from the previous angioedema attack, the new symptoms must occur at least 24 h after resolution of the symptoms of the prior angioedema attack.

The efficacy of lanadelumab will be evaluated using the angioedema attack data during the treatment period (Day 0 through Day 364). Efficacy end points will include the following:

- Time to first attack for rollover patients
- Number of investigator-confirmed attacks
- Number of investigator-confirmed attacks requiring acute treatment
- Number of moderate and severe attacks. Attack severity will be assessed as mild (transient or mild discomfort), moderate (mild to moderate limitation in activity; some assistance required) or severe (marked limitation in activity; assistance required).
- Number of high-morbidity attacks, which are defined as any attack with at least one of the following characteristics: severe, results in hospitalization, hemodynamically significant or upper airway (laryngeal).

Blood samples will be collected at various time points during the study to measure the following:

- Plasma lanadelumab concentrations to obtain a pharmacokinetic profile [7]
- Pharmacodynamic biomarkers (plasma kallikrein activity, as measured by cleaved HMWK levels) [7]
- Presence of anti-drug antibodies. Samples that are confirmed positive for anti-drug antibodies will be further analyzed for neutralizing antibodies. The method of analysis has been previously described [7]
- Functional C1-INH, C4, and C1q levels for eligibility assessment at screening for non-rollover patients.

Patient quality of life will be assessed using the following:

- Angioedema Quality of Life Questionnaire (AE-QoL) [10]
- EuroQol 5-Dimensional 5-Level Measure (EQ-5D-5L) [11]
- Work Productivity and Activity Impairment Questionnaire: General Health (WPAI-GH) [12]
- Hospital Anxiety and Depression Scale (HADS) [13]
- 12 Item Short Form Survey (SF-12) [14]

If applicable, patients will be surveyed on their experience with self-administration and subcutaneous injection of lanadelumab every 6 months during the study. Patients who previously received long-term prophylaxis with C1-INH products by intravenous administration will be asked to indicate the preferred route for medication administration.

### Sample size

The sample size for this single arm, open-label study is not based on a formal statistical sample size calculation. This study does not have a control arm, thus no formal statistical hypothesis testing will be performed. All p-values will be considered descriptive.

### Individual stopping rules

Dosing for any individual patient will be discontinued if the patient experiences a serious adverse event or clinically significant non-serious adverse event that is related to lanadelumab treatment and that, in the opinion of the investigator or the independent Data and Safety Monitoring Board, warrants discontinuation from further dosing for that patient’s well-being. Patients who discontinue from the study will undergo, if possible, all assessments and procedures scheduled for Day 378–392 (see Additional file 1: Table S1).

### Treatment compliance

All doses of open-label lanadelumab administered at the investigational site will be given under the direct supervision of the investigator or designee. Doses that are self-administered off site will be confirmed by site personnel within approximately 3 days of the planned administration. Patients will be contacted to ensure the administration occurred, to collect information on adverse events and concomitant medications, and to ensure all attacks have been appropriately documented.

### Data analysis

The safety population will include all patients who received any study drug after entering the open-label extension study. The rollover safety population is the subset of patients who participated in the double-blind study and received any study drug after entering the open-label extension study. The non-rollover safety population is the subset of patients who directly entered the open-label extension study and subsequently received any study drug.

All available data will be included in the analysis. No imputation of missing data will be performed. Summary tabulations conducted with the non-rollover safety population will be presented by patient’s type of LTP prior to study entry.

AEs will be coded using the Medical Dictionary for Regulatory Activities and will be summarized by system organ class and preferred term for each population. The number of events and the number and percentage of patients with any treatment-emergent adverse event, serious adverse event, severe adverse event, or adverse event of special interest will be summarized according to relatedness to treatment.

Actual values and change from baseline in clinical laboratory test results and vital signs will be summarized by study visit. The number and percentage of patients with normal, abnormal, and abnormal clinically significant electrocardiogram results will be summarized by study visit.

Time to the first investigator-confirmed attack will be analyzed using the rollover safety population. Patients who discontinue the study prior to experiencing their first confirmed attack will be censored at the date and time of discontinuation. Data will be summarized using Kaplan–Meier methods. The number of angioedema attacks from Day 0 through Day 364 will be expressed as a monthly angioedema attack rate and will be analyzed for each analysis population. The monthly attack rate will be calculated for each patient as the number of attacks occurring during the treatment period divided by the number of days the patient contributed to the treatment period multiplied by 28 days. The attack rate will also be analyzed by subgroups, including age group, sex, race, weight, body mass index, baseline angioedema attack rate, HAE type, geographic region, and administration type. Sensitivity analyses will be performed on the attack rate to evaluate the robustness of the results.

Plasma lanadelumab concentrations and plasma kallikrein activity endpoints will be summarized by nominal sampling times. The number and percentage of patients with positive antibodies will be summarized by study visit and overall.

Quality of life assessments will be summarized by study visit.

## Discussion

This study will provide important information on the long-term safety and efficacy of lanadelumab in preventing angioedema attacks. An evaluation of lanadelumab over an extended period is valuable, as real-world use of lanadelumab would involve ongoing, repeated dosing. To date, clinical studies for lanadelumab have not indicated any safety concerns and have suggested preliminary data for efficacy in preventing angioedema attacks in patients with HAE. In a Phase 1a study, 32 healthy volunteers received a single dose of lanadelumab (dose range 6.2–302 mg) or placebo [6]. All doses were well tolerated, there were no deaths, serious adverse events, or discontinuations owing to adverse events, and treatment-emergent adverse events were comparable between subjects who received lanadelumab and those who received placebo. None of the subjects were positive for anti-drug antibodies. In a Phase 1b study, patients with HAE received two doses of lanadelumab (30, 100, 300 or 400 mg) or placebo, administered 14 days apart. The rate of adverse events was similar in lanadelumab-treated patients compared with those who received placebo, and the results of other safety outcome measures showed that lanadelumab was safe up to 400 mg. Non-neutralizing anti-drug antibodies were detected in a total of two patients: one in the 30 mg dose group, and one in the 400 mg dose group. Furthermore, the attack rate was reduced by 100 and 88% in patients who received 300 mg and 400 mg lanadelumab, respectively, relative to placebo. The reduction in attack rate coincided with drug exposure and a decrease in cleaved HMWK levels, providing proof of concept for the mechanism of action of lanadelumab [7].

The double-blind study will assess the efficacy and safety of lanadelumab over 26 weeks of treatment. Following the double-blind study, results from the open-label extension study will confirm findings on safety, efficacy, and pharmacodynamic effect from prior clinical studies. By delaying the second dose for rollover patients until the first attack has occurred, the study design incorporates a unique feature to investigate the pharmacodynamic effect of lanadelumab. However, the design is not adaptable in that individual adjustments in the dosing interval will not be made based upon each patient’s response. The open-label extension study will also assess the safety, efficacy, and impact on quality of life of lanadelumab for patients who are currently using other long-term prophylactic therapies. Patients with HAE have a strong preference for self-administration of therapeutics [15, 16], and the open-label extension study will evaluate patients’ experience with self-administration of lanadelumab. This will help to assess treatment burden and potential ease and convenience of lanadelumab therapy.

A recognized potential limitation of this study, as for any controlled clinical trial with established inclusion and exclusion criteria for enrollment, is the possibility of investigator bias introduced by study patient selection. Some degree of selection bias may inherently be introduced as part of the study enrollment process. These potential limitations will be acknowledged and addressed as part of the Discussion when results are available.

Although current prophylactic treatments for HAE may be effective at reducing the frequency, duration, and severity of HAE attacks, there remains an unmet need for more effective and less burdensome treatment of attacks. The key attributes for a new prophylactic therapy would be improved efficacy (reduced attack frequency and increased proportion of patients who are attack-free), safety and tolerability, and patient convenience in order to ease both the burden of illness and treatment for individuals with HAE.

## Additional file

**Additional file 1: Table S1.** Study activities.

## Abbreviations

C1-INH: C1 inhibitor
HAE: hereditary angioedema
HMWK: high molecular weight kininogen
LTP: long-term prophylaxis
