# Supplementary material for: An open-label study to evaluate the long-term safety and efficacy of lanadelumab for prevention of attacks in hereditary angioedema: design of the HELP study extension
Source: Clin Transl Allergy. 2017 Oct 6;7:36. doi: 10.1186/s13601-017-0172-9 (PMC5629784; doi:10.1186/s13601-017-0172-9)
Supplement: Supplementary file 1 — Additional file 1: Table S1. Study activities. [file 13601_2017_172_MOESM1_ESM.pdf]

Table S1. Study Activities

Tests and assessments		Treatment period ±4 days for each visit														
**Non-rollover**	**Screen visit[1]**															
**Rollover**	-		**Check-in[2]**													
**Visit:**		**1**		**2**	**3**	**4**	**5**	**6**	**7**	**8**	**9**	**10**	**11**	**12**	**13**	**14**
**Dose:**		**1**		**2**	**3**	**4**	**5**	**6**	**7**	**8**	**9**	**10**	**11**	**12**	**13**	**14**
**Day:**		**0**		**14**	**28**	**42**	**56**	**70**	**84**	**98**	**112**	**126**	**140**	**154**	**168**	**182**
Informed consent[5]	X	(X)[6]														
Eligibility review	X	X														
LTP therapy continued[7]	X	X		X												
Lanadelumab administration (rollover)		X		(X)[8,9]	(X)[10]	(X)[11]	(X)[10]	(X)[11]	(X)[11]	(X)[10]	(X)[11]	(X)[10]	(X)[11]	(X)[10]	(X)[11]	(X)[10]
Lanadelumab administration (non-rollover)		X		X	X[10]	X[11]	X[10]	X[11]	X[11]	X[10]	X[11]	X[10]	X[11]	X[10]	X[11]	X[10]
Demographic and medical history	X															
C1-INH, C1q and C4 testing[12]	X															
Pregnancy test[13] (females)	X	X		X	X		X			X		X		X		X
Vital signs[14]	X	X		X	X	X	X	X	X	X	X	X	X	X	X	X
Physical exam[15]	X	X		X	X		X			X				X		X
Clinical laboratory testing[16]	X	X			X		X			X				X		X
12-Lead ECG	X	X								X						X
Prior (4 weeks) and concomitant therapy	X	X	X	X	X	X	X	X	X	X	X	X	X	X	X	X
Adverse events	X	X	X	X	X	X	X	X	X	X	X	X	X	X	X	X
HAE attack data[17]	X	X	X	X	X	X	X	X	X	X	X	X	X	X	X	X
Quality of life assessments[18]		X			X		X			X		X		X		X
Lanadelumab injection report[19]		X		X	X	X	X	X	X	X	X	X	X	X	X	X
Lanadelumab self-administration and subcutaneous injection survey[20]	X									X						X
PK, PD collection and plasma antibody testing[21]	X									X						X
Discharge from study																

C1-INH C1 inhibitor; *DB* double-blind; *ECG* electrocardiogram; *HAE* hereditary angioedema; *LTP* long-term prophylaxis; *OLE* open-label extension; *PD* pharmacodynamic; *PK* pharmacokinetic

Shaded columns represent scheduled on-site visits for all patients. Non-shaded columns indicate potential patient-elected off-site activity and/or self-administration dosing

Parentheses indicate activities that may occur as applicable (ie, activities for rollover patients)

1. Screening visit is for non-rollover patients only. Screening visit can occur up to 28 days prior to first open-label dose
2. Study site personnel will contact rollover patients approximately every 7 days following the first dose of open-label lanadelumab to solicit for any HAE attacks not already reported. Site check-in with rollover patients will continue until the patient receives their second open-label dose
3. Visit 28 is a site check-in call for all rollover and non-rollover patients
4. Patients who terminate the study early will undergo (if possible) all of the assessments and procedures at visit 29, the final study visit
5. Rollover patients must sign informed consent for the OLE study on or after Day 168 of the DB study
6. For rollover patients Day 182 of the DB study is also Day 0 of the OLE study and informed consent may be completed on this visit, if not already provided
7. Screened non-rollover patients (adults and adolescents) who are on LTP with C1-INH therapy for HAE can continue their current LTP until Day 8 (or Day 15) such that patients will have received two (or four) doses of C1-INH. For patie who are on attenuated androgens or anti-fibrinolytics, a similar tapering schedule is recommended. However, the taper can be prolonged by an additional week as long as androgen or anti-ifibrinolytic therapy is stopped within 3 weeks of receiving the first dose of lanadelumab
8. For rollover patients, the timing of dose 2 for will vary by patient based on when their first HAE attack occurs following dose 1. Following the first reported and investigator-confirmed attack, patients will begin receiving regular subcutaneous administrations of 300 mg lanadelumab every 2 weeks
9. A minimum of 10 days between the first and second open-label doses is required. If the second dose is to be administered within the accepted ±4-day window around a scheduled study visit, this treatment visit will represent that scheduled visit unless that scheduled visit has already occurred. If that scheduled visit has already occurred, or if the second dose is to be administered outside of the accepted ±4-day window around a scheduled visit, this visit will not replace any scheduled visit and will thus represent an acceptable, extra study visit (ie, an unscheduled visit). Regardless, at the visit in which the second open-label dose of lanadelumab is administered, the patient will undergo pre-dose assessments for vital signs, physical examination, clinical laboratory testing, PK, PD and anti-drug antibody. Vital signs will be obtained at 1 hour post dosing. As with all study visits, information will be collected on adverse events, concomitant therapy, and HAE attack data
10. All patients (adolescent or adult) who are considered suitable candidates (ie, those with a physical and mental capability for learning and willing to be trained) may be allowed to self-administer lanadelumab after completing appropriate training by the investigator or designee and confirming their understanding. Patients are allowed to initiate home self-administration after receiving the first two doses of lanadelumab at the study site and may elect to self-administer subsequent doses of lanadelumab at the investigational site (during scheduled study site visits; shaded columns)
11. Patients may self-administer lanadelumab at home or other agreed upon location (during optional off-site self-administration visits; non-shaded columns). Site personnel will call patients within approximately 3 days of the planned self-administration to ensure the administration occurred, to collect AEs and concomitant medications, and to ensure all attacks have been appropriately documented
12. Samples for C1-INH, C4 and C1q assays will be obtained at screening for eligibility assessment unless already collected as part of the Phase 1b study (DX-2930-02; NCT02093923) or the DB study
13. The pregnancy test will only be conducted in females of childbearing potential.
14. Vital signs will be obtained prior to dosing and 1 hour after dosing. Monitoring of vital signs will not be performed for patients who elect to self-administer away from the investigative site at optional off-site visits (indicated non-shaded columns)
15. Physical examinations, including weight, will be conducted for all rollover and non-rollover patients according to the study activities schedule and in accordance with standards at the site. In addition to the physical examinations specified in the study activities schedule, an additional physical examination (performed in accordance with standards at the site) will be conducted for rollover patients prior to dosing on the day of their second open-label dose, at whatever study visit that occurs. Height will be collected at the screening visit only
16. Clinical laboratory testing will include hematology, coagulation, serum chemistry and urinalysis (urinalysis does not need to be done as part of the clinical laboratory testing at visits 14, 17, 20 and 23). Clinical laboratory testing will be conducted for all rollover and non-rollover patients according to the study activities schedule. In addition to the testing specified in the study activities schedule, additional testing will be conducted for rollover patients prior to dosing on the of their second open-label dose, at whatever study visit that occurs
17. Historical HAE attack information will be collected at screening. During the study, patients (or caregivers) are instructed to report details of the attack to the study site within 72 hours of the onset of the attack. During study visits, study site personnel will solicit for any new HAE attack information that has not already been reported to the site
18. Quality of life data will be obtained using the EQ-5D-5L, SF-12, AE-QoL, HADS and WPAI-GH
19. Collect patient's injection reports of their experience with lanadelumab self-administration and subcutaneous administration for all doses
20. Collect patient's injection surveys of their experience with lanadelumab self-administration and subcutaneous injection for indicated visits
21. PK, PD and anti-drug antibody samples will be drawn for all rollover and non-rollover patients according to the study activities schedule. In addition to the samples specified in the study activities schedule, an additional PK, PD and anti-drug antibody sample will be drawn for rollover patients prior to dosing on the day of their second open-label dose, at whatever study visit that occurs

Table S1. Study Activities

Tests and assessments	Treatment period ±4 days for each visit													Follow-up ±4 days for each visit	
Non-rollover															
Rollover															
**Visit:**	**15**	**16**	**17**	**18**	**19**	**20**	**21**	**22**	**23**	**24**	**25**	**26**	**27**	**28** [3]	**29** [4]
**Dose:**	**15**	**16**	**17**	**18**	**19**	**20**	**21**	**22**	**23**	**24**	**25**	**26**	**-**	**-**	**-**
**Day:**	**196**	**210**	**224**	**238**	**252**	**266**	**280**	**294**	**308**	**322**	**336**	**350**	**364**	**378**	**392**
Informed consent[5]															
Eligibility review															
LTP therapy continued[7]															
Lanadelumab administration (rollover)	(X)[11]	(X)[11]	(X)[10]	(X)[11]	(X)[11]	(X)[10]	(X)[11]	(X)[11]	(X)[10]	(X)[11]	(X)[11]	(X)[10]			
Lanadelumab administration (non-rollover)	X[11]	X[11]	X[10]	X[11]	X[11]	X[10]	X[11]	X[11]	X[10]	X[11]	X[11]	X[10]			
Demographic and medical history															
C1-INH, C1q and C4 testing[12]															
Pregnancy test[13] (females)			X			X			X			X	X		X
Vital signs[14]	X	X	X	X	X	X	X	X	X	X	X	X	X		X
Physical exam[15]						X						X	X		X
Clinical laboratory testing[16]			X			X			X			X	X		X
12-Lead ECG						X						X	X		X
Prior (4 weeks) and concomitant therapy	X	X	X	X	X	X	X	X	X	X	X	X	X	X	X
Adverse events	X	X	X	X	X	X	X	X	X	X	X	X	X	X	X
HAE attack data[17]	X	X	X	X	X	X	X	X	X	X	X	X	X	X	X
Quality of life assessments[18]			X			X			X			X			X
Lanadelumab injection report[19]	X	X	X	X	X	X	X	X	X	X	X	X			
Lanadelumab self-administration and subcutaneous injection survey[20]						X						X			
PK, PD collection and plasma antibody testing[21]						X							X		X
Discharge from study															X

C1-INH C1 inhibitor; *DB* double-blind; *ECG* electrocardiogram; *HAE* hereditary angioedema; *LTP* long-term prophylaxis; *OLE* open-label extension; *PD* pharmacodynamic; *PK* pharmacokinetic

Shaded columns represent scheduled on-site visits for all patients. Non-shaded columns indicate potential patient-elected off-site activity and/or self-administration dosing

Parentheses indicate activities that may occur as applicable (ie, activities for rollover patients)

1. Screening visit is for non-rollover patients only. Screening visit can occur up to 28 days prior to first open-label dose
2. Study site personnel will contact rollover patients approximately every 7 days following the first dose of open-label lanadelumab to solicit for any HAE attacks not already reported. Site check-in with rollover patients will continue until the patient receives their second open-label dose
3. Visit 28 is a site check-in call for all rollover and non-rollover patients
4. Patients who terminate the study early will undergo (if possible) all of the assessments and procedures at visit 29, the final study visit
5. Rollover patients must sign informed consent for the OLE study on or after Day 168 of the DB study
6. For rollover patients Day 182 of the DB study is also Day 0 of the OLE study and informed consent may be completed on this visit, if not already provided
7. Screened non-rollover patients (adults and adolescents) who are on LTP with C1-INH therapy for HAE can continue their current LTP until Day 8 (or Day 15) such that patients will have received two (or four) doses of C1-INH. For patients who are on attenuated androgens or anti-fibrinolytics, a similar tapering schedule is recommended. However, the taper can be prolonged by an additional week as long as androgen or ant-ifibrinolytic therapy is stopped within 3 weeks of receiving the first dose of lanadelumab
8. For rollover patients, the timing of dose 2 for will vary by patient based on when their first HAE attack occurs following dose 1. Following the first reported and investigator-confirmed attack, patients will begin receiving regular subcutaneous administrations of 300 mg lanadelumab every 2 weeks
9. A minimum of 10 days between the first and second open-label doses is required. If the second dose is to be administered within the accepted ±4-day window around a scheduled study visit, this treatment visit will represent that scheduled visit unless that scheduled visit has already occurred. If that scheduled visit has already occurred, or if the second dose is to be administered outside of the accepted ±4-day window around a scheduled visit, this visit will not replace any scheduled visit and will thus represent an acceptable, extra study visit (ie, an unscheduled visit). Regardless, at the visit in which the second open-label dose of lanadelumab is administered, the patient will undergo pre-dose assessments for vital signs, physical examination, clinical laboratory testing, PK, PD and anti-drug antibody. Vital signs will be obtained at 1 hour post dosing. As with all study visits, information will be collected on adverse events, concomitant therapy, and HAE attack data
10. All patients (adolescent or adult) who are considered suitable candidates (ie, those with a physical and mental capability for learning and willing to be trained) may be allowed to self-administer lanadelumab after completing appropriate training by the investigator or designee and confirming their understanding. Patients are allowed to initiate home self-administration after receiving the first two doses of lanadelumab at the study site and may elect to self-administer subsequent doses of lanadelumab at the investigational site (during scheduled study site visits; shaded columns)
11. Patients may self-administer lanadelumab at home or other agreed upon location (during optional off-site self-administration visits; non-shaded columns). Site personnel will call patients within approximately 3 days of the planned self-administration to ensure the administration occurred, to collect AEs and concomitant medications, and to ensure all attacks have been appropriately documented
12. Samples for C1-INH, C4 and C1q assays will be obtained at screening for eligibility assessment unless already collected as part of the Phase 1b study (DX-2930-02; NCT02093923) or the DB study
13. The pregnancy test will only be conducted in females of childbearing potential.
14. Vital signs will be obtained prior to dosing and 1 hour after dosing. Monitoring of vital signs will not be performed for patients who elect to self-administer away from the investigative site at optional off-site visits (indicated non-shaded columns)
15. Physical examinations, including weight, will be conducted for all rollover and non-rollover patients according to the study activities schedule and in accordance with standards at the site. In addition to the physical examinations specified in the study activities schedule, an additional physical examination (performed in accordance with standards at the site) will be conducted for rollover patients prior to dosing on the day of their second open-label dose, at whatever study visit that occurs. Height will be collected at the screening visit only
16. Clinical laboratory testing will include hematology, coagulation, serum chemistry and urinalysis (urinalysis does not need to be done as part of the clinical laboratory testing at visits 14, 17, 20 and 23). Clinical laboratory testing will be conducted for all rollover and non-rollover patients according to the study activities schedule. In addition to the testing specified in the study activities schedule, additional testing will be conducted for rollover patients prior to dosing on the day of their second open-label dose, at whatever study visit that occurs
17. Historical HAE attack information will be collected at screening. During the study, patients (or caregivers) are instructed to report details of the attack to the study site within 72 hours of the onset of the attack. During study visits, study site personnel will solicit for any new HAE attack information that has not already been reported to the site
18. Quality of life data will be obtained using the EQ-5D-5L, SF-12, AE-QoL, HADS and WPAI-GH
19. Collect patient’s injection reports of their experience with lanadelumab self-administration and subcutaneous administration for all doses
20. Collect patient’s injection surveys of their experience with lanadelumab self-administration and subcutaneous injection for indicated visits
21. PK, PD and anti-drug antibody samples will be drawn for all rollover and non-rollover patients according to the study activities schedule. In addition to the samples specified in the study activities schedule, an additional PK, PD and anti-dı antibody sample will be drawn for rollover patients prior to dosing on the day of their second open-label dose, at whatever study visit that occurs
